# Proximal Migration of Pancreatic Duct Stent in Pancreas Divisum: Challenges in Retrieval and Review of the Literature

**DOI:** 10.1155/2021/5531658

**Published:** 2021-04-20

**Authors:** Subash Ghimire, Shri Jaikishan Ravi, Mohammad Yousef, Hafiz Khan

**Affiliations:** Guthrie Robert Packer Hospital, Sayre, PA 18840, USA

## Abstract

Pancreas divisum is the most common congenital malformation of the pancreas. Sometimes it is considered an etiology when patients present with recurrent episodes of pancreatitis. Endoscopic retrograde pancreatography (ERP) with selective cannulation of the minor papilla with sphincterotomy and stent placement are performed in these patients. Proximal migration of pancreatic stents in pancreas divisum is rare and challenging to manage. We describe a case of proximal migration of a pancreatic stent in a patient with pancreas divisum and perform a review of literature.

## 1. Introduction

Pancreas divisum is the most common congenital malformation of the pancreas, occasionally implicated as a cause of idiopathic recurrent pancreatitis [1]. Diagnosis of pancreas divisum can be made by magnetic resonance cholangiopancreatography (MRCP), endoscopic ultrasound (EUS), or ERP. If symptomatic, these patients undergo endoscopic retrograde pancreatography (ERP) with sphincterotomy of the minor papilla and stent placement [2]. Proximal migration of the pancreatic stent is uncommon, and its retrieval can be challenging. The retrieval of large pancreatic duct (PD) stones is usually achieved by stone fragmentation using electrohydraulic or laser lithotripsy and extracorporeal shock wave lithotripsy [3]. We describe a case of 55-year-old female with pancreas divisum and pancreatic duct stones in the dorsal duct who had undergone pancreatic duct stent placement for recurrent pancreatitis complicated by proximal stent migration.

## 2. Case Presentation

A 55-year-old female with a past medical history of pancreas divisum, recurrent pancreatitis, diabetes mellitus, and hypertension presented with sharp epigastric pain radiating to her back associated with nausea and vomiting. On physical exam, she appeared stable with moderate epigastric tenderness. Lab work including liver enzymes and lipase were in normal range. Cross-sectional imaging revealed pancreas divisum and multiple stones in the dorsal pancreatic duct. Because of her recurrent symptoms, ERP with planned selective cannulation of the minor papilla was performed.

**Figure 1:**
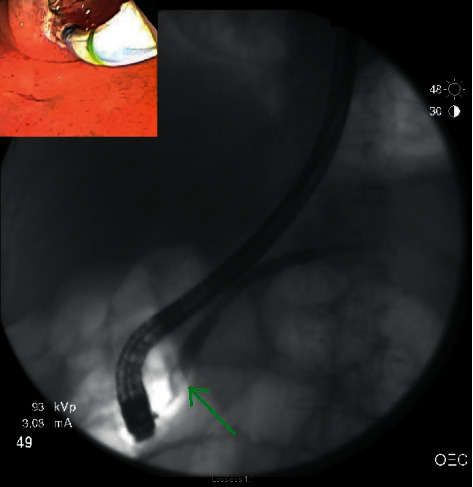
Selective cannulation of minor papilla and stone in the pancreatic duct.

**Figure 2:**
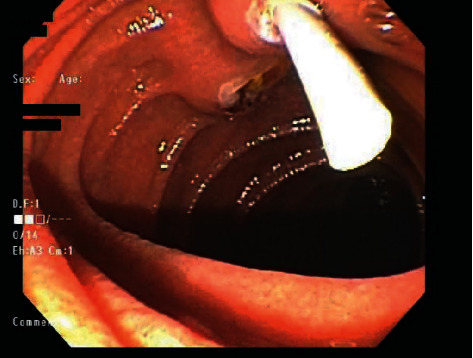
Plastic stent placed into the main pancreatic duct.

ERP revealed a stenosed minor papilla, which was selectively cannulated, and sphincterotomy was performed (Figure 1). Main pancreatic duct appeared dilated to 8 mm, and few stones were seen on contrast injection as shown in Figure 1. One stone was removed, and an 8.5 Fr x 9 cm straight plastic stent was placed into the main pancreatic duct (Figure 2). After the procedure, she had adequate relief of symptoms and was discharged home.

**Figure 3:**
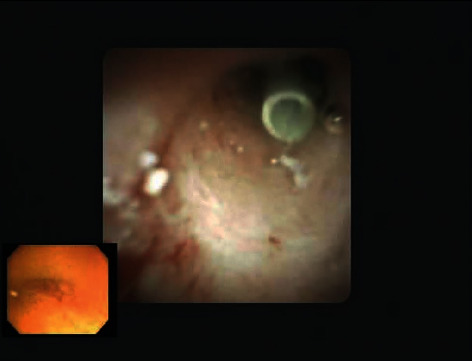
Showing migrated Pancreatic duct stent.

**Figure 4:**
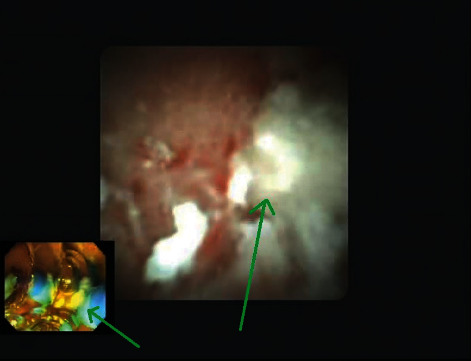
SpyGlass view and removal of PD stones.

ERP was performed 3 weeks later for the removal of PD stones and a previously placed plastic stent. The stent was found to have migrated deep into the main pancreatic duct (Figure 3). The pancreatic duct was explored endoscopically using SpyGlass Direct Visualization System (Boston Scientific, Natick, Massachusetts, USA). Multiple stones were seen causing obstruction of the duct, and these stones were removed using a spy basket through SpyGlass pancreatoscope (Figure 4). After ductal clearance was achieved, a proximally migrated PD stent was seen, which was successfully retrieved using a SpyGlass Retrieval Snare under direct visualization. A 5 Fr x 9 cm single pigtail pancreatic stent was placed to ensure adequate drainage after intraductal intervention and was removed after 1 week. The patient had significant improvement in her symptoms after ductal clearance and minor papilla sphincterotomy and has not required hospitalization in one year.

## 3. Discussion

Pancreas divisum is a common congenital condition of the pancreas [2]. It is usually an incidental finding, most of the patients remain asymptomatic, and less than 5% can develop pancreatitis. Mild pancreatitis can be managed conservatively, but severe and recurrent episodes require ERP with minor papilla sphincterotomy to facilitate pancreatic ductal drainage. During this procedure, a stent may be placed in the main pancreatic duct to maintain the patency of the duct when a sphincterotomy is performed [4].

Pancreatic stents can be metallic or plastic. Plastic stents are used more often and are made up of polyethylene. They are 2 to 25 cm in length, and the diameter varies from 3 French to 11.5 French. Pancreatic stents can be shaped as straight, curved, wedged, or with a single pigtail. If prolonged stenting is required, stents with internal flanges are used to prevent distal migration [5].

Complications associated with pancreatic stent placement are abdominal pain, stent obstruction, pancreatitis, disruption of the pancreatic duct mucosa, and bleeding. Complications from the pancreatic stent itself include dislocation, fracture, and migration of the stent. [2, 4].

Pancreatic stents can migrate both distally as well as proximally into the pancreatic duct. Migrated stents can cause recurrent pancreatitis, infection, perforation, as well as stenosis of the duct. Incidence of stent migration has been reported at about 1.5% in the medical literature [6]. Retrieval of the migrated stent is challenging, especially in pancreas divisum due to the anatomical location of the main pancreatic duct and the acute angle at which the endoscope must be maneuvered. Use of alternate techniques, including basket catheter and metallic spiral stent retriever, has been described in literature. If the tip of the migrated stent is seen in major or minor papilla, direct traction can be used. If the migrated stent cannot be seen, balloon catheter can be used to extract the stent, and stent can be pulled using forceps [7, 8]. Use of SpyGlass Direct Visualization System enables direct and fluoroscopic visualization of the pancreatic duct. Success rate of endoscopic retrieval is estimated from 70% to 100% [8]. If endoscopic retrieval is not possible, surgical retrieval may be necessary [6].

Previous studies have described stent migration in patients with prolonged stent placement or in patients who require frequent stent exchanges. Horwhat et al. have postulated that prior sphincterotomy might increase the risk of stent migration [9]. Hogan et al. have postulated multiple risk factors for proximal migration of the stent including cholangiocarcinoma, malignant strictures, and longer length of the stent used (if it traverses the genu of the pancreas) [2]. Most likely risk factors in our case for proximal stent migration were minor papilla sphincterotomy, use of long straight plastic stent without internal flanges, and probably lack of angulation in the dorsal duct in pancreas divisum with multiple intraductal stones.

## 4. Conclusion

There is a paucity of data on stent migration and retrieval from the main pancreatic duct in pancreas divisum. Location as well as varying anatomical structure of the minor papilla and main pancreatic duct makes stent retrieval challenging. Our case highlights the success in retrieving migrated pancreatic duct stent and pancreatic duct stones using SpyGlass Direct Visualization System in patients with pancreas divisum.
